# Mercury in the human adrenal medulla could contribute to increased plasma noradrenaline in aging

**DOI:** 10.1038/s41598-021-82483-y

**Published:** 2021-02-03

**Authors:** Roger Pamphlett, Stephen Kum Jew, Philip A. Doble, David P. Bishop

**Affiliations:** 1 Discipline of Pathology, Sydney Medical School, Brain and Mind Centre, The University of Sydney, Sydney, NSW Australia; 2 Department of Neuropathology, Royal Prince Alfred Hospital, Sydney, NSW Australia; 3 Elemental Bio-Imaging Facility, School of Mathematical and Physical Sciences, University of Technology Sydney, Sydney, NSW Australia

**Keywords:** Endocrine system and metabolic diseases, Hypertension, Environmental impact, Metals, Hormones, Adrenal cortex hormones, Adrenal gland diseases, Metabolic syndrome

## Abstract

Plasma noradrenaline levels increase with aging, and this could contribute to the sympathetic overactivity that is associated with essential hypertension and the metabolic syndrome. The underlying cause of this rise in noradrenaline is unknown, but a clue may be that mercury increases noradrenaline output from the adrenal medulla of experimental animals. We therefore determined the proportion of people from 2 to 104 years of age who had mercury in their adrenal medulla. Mercury was detected in paraffin sections of autopsied adrenal glands using two methods of elemental bioimaging, autometallography and laser ablation-inductively coupled plasma-mass spectrometry. Mercury first appeared in cells of the adrenal medulla in the 21–40 years group, where it was present in 52% of samples, and increased progressively in frequency in older age groups, until it was detected in 90% of samples from people aged over 80 years. In conclusion, the proportion of people having mercury in their adrenal medulla increases with aging. Mercury could alter the metabolism of catecholamines in the adrenal medulla that leads to the raised levels of plasma noradrenaline in aging. This retrospective autopsy study was not able to provide a definitive link between adrenal mercury, noradrenaline levels and hypertension, but future functional human and experimental studies could provide further evidence for these associations.

## Introduction

Plasma noradrenaline increases with age^1^, and increased noradrenaline activity is thought to play a role in the pathogenesis of essential hypertension^2–4^ and the metabolic syndrome^5^. One proposed mechanism for the increased noradrenaline levels during aging is overactivity of central nervous system sympathetic neurons, followed by a spillover of noradrenaline into the plasma from postganglionic neurons^4,6^, but the cause of this central neuronal overactivity is obscure. In contrast to elevated noradrenaline, adrenaline secretion from the adrenal medulla decreases with age^6^. The cause of this decreased adrenaline output remains unknown.

**Figure 1:**
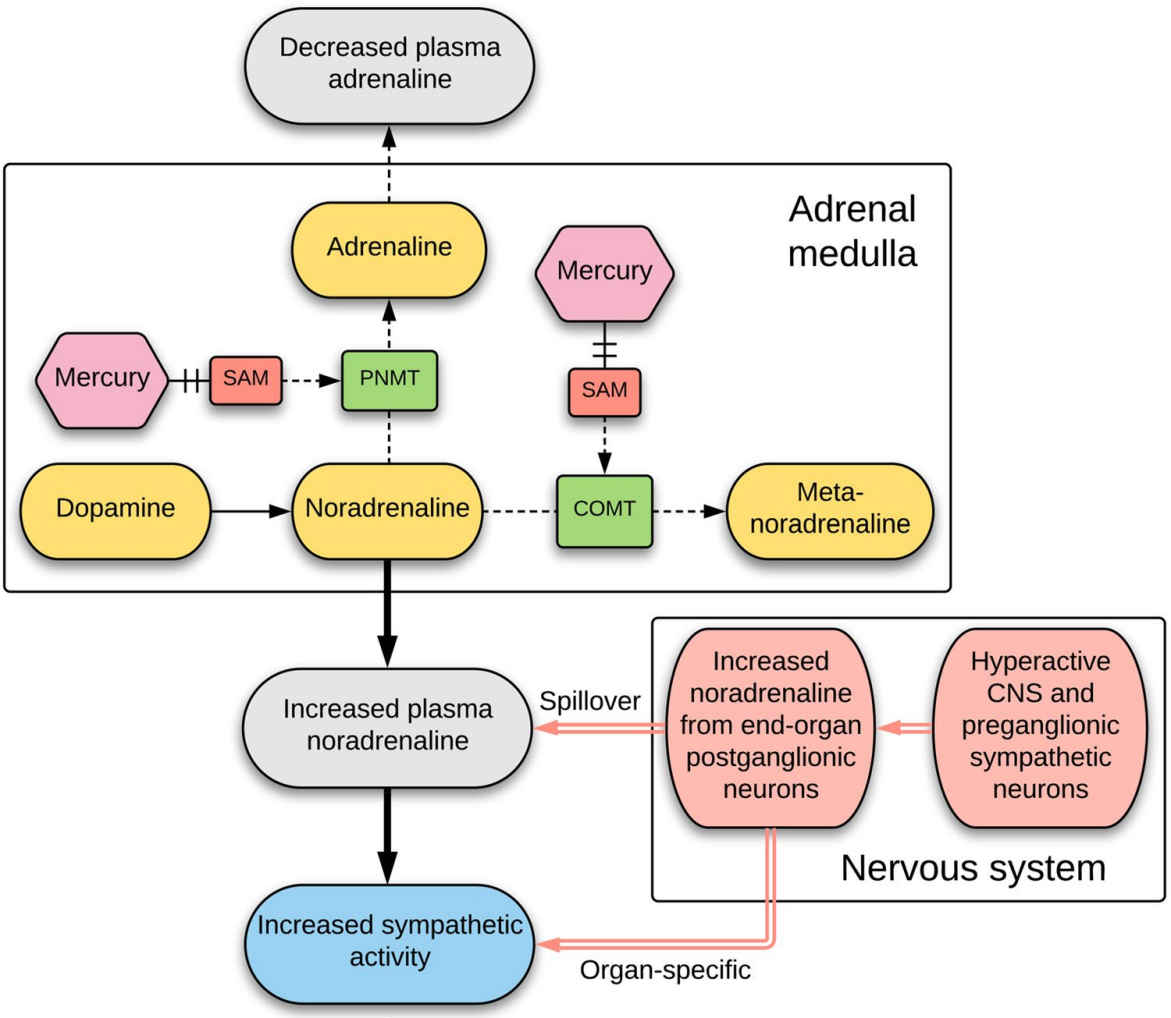
Pathways for mercury-induced increased noradrenaline and decreased adrenaline output. Mercury, by binding to and inhibiting the cofactor S-adenosyl-l-methionine (SAM) in the adrenal medulla, reduces the ability of catechol-*O*-methyltransferase (COMT) to deactivate noradrenaline, resulting in an increased output of noradrenaline. Mercury binding to SAM also reduces the ability of phenylethanolamine *N*-methyltransferase (PNMT) to convert noradrenaline into adrenaline, resulting in a decreased output of adrenaline. Overactivity of central nervous system sympathetic neurons (of unknown cause) may contribute to increased sympathetic activity via elevated noradrenaline output from postganglionic neurons.

Another possible origin for the elevated noradrenaline in aging is the uptake of toxic metals by the adrenal medulla. Exposures to toxic metals, such as mercury and cadmium, release noradrenaline from the adrenal medulla of experimental animals^7,8^. Several case studies, mostly in children, have reported acute hypertension after accidental exposure to mercury^9–16^. Mercury in the adrenal medulla has been proposed to deactivate the cofactor S-adenosylmethionine (SAM), due to its high number of mercury-binding sulfhydryl groups^12^. This would decrease the activity of two SAM-dependent enzymes, catechol-*O*-methyltransferase that deactivates noradrenaline, and phenylethanolamine *N*-methyltransferase that converts noradrenaline into adrenaline (Fig. 1). The result would be an increased output of noradrenaline and a decreased secretion of adrenaline, the situation found in human aging. This mechanism provides a potential link between the findings, in both essential hypertension and the metabolic syndrome, of increased sympathetic activity^4,5^ and previous exposure to mercury^17,18^. It may also underlie the acutely-raised noradrenaline levels and hypertension that follow accidental exposure to mercury^14^.

Selective uptake of mercury by the adrenal medulla has been demonstrated in rodents^19,20^ and primates^21,22^, but it is not known if mercury is taken up by the human adrenal medulla. To see if mercury in the adrenal medulla could contribute to the changes in catecholamine levels found in aging, we looked for the presence of mercury and other toxic metals in the adrenal glands of people aged between 2 and 104 years.

## Methods

### Sample collection

Paraffin-embedded samples of adrenal glands were examined from 89 individuals who had autopsies performed in the New South Wales Department of Forensic Medicine, Sydney (Supplementary Table S1). Ages ranged between 2 and 104 years, and comprised 56 males (mean age 52 years, SD 26 years, range 2–98 years) and 33 females (mean age 59 years, SD 29 years, range 2–104 years). Clinical histories were: no major known disorder (N = 36), neurodegenerative disorder (N = 26), psychosis (N = 22), two with epilepsy, and one each of post-traumatic stress disorder, Down syndrome, and anorexia nervosa. Causes of death were: suicide (N = 23), trauma (N = 15), cardiovascular (N = 14), drug overdose (N = 10), drowning (N = 8), choking (N = 4), infection (N = 4), undetermined (N = 4), cerebrovascular (N = 2), respiratory (N = 2), and one each of cancer, cirrhosis, and undernutrition.

### Autometallography

Seven-micron paraffin sections of the adrenal medulla were stained for inorganic mercury bound to sulphide or selenide using silver nitrate autometallography (AMG), which represents the presence of mercury as black grains^23^. Briefly, sections were placed in physical developer containing 50% gum arabic, citrate buffer, hydroquinone and silver nitrate at 26 °C for 80 min in the dark then washed in 5% sodium thiosulphate to remove unbound silver. Sections were counterstained with mercury-free hematoxylin and viewed with bright-field microscopy using an Olympus BX50 microscope. Each staining run included a control section of mouse spinal cord where motor neuron cell bodies contained mercury following an intraperitoneal injection of mercuric chloride^24^. The proportion of adrenal chromaffin cells containing AMG was categorised as ‘low’ if AMG was present in up to 25% of cells, and ‘high’ if AMG was present in more than 25% of cells in at least four 200× microscopic fields.

### Laser ablation-inductively coupled plasma-mass spectrometry (LA-ICP-MS)

In addition to mercury, AMG detects the presence of inorganic silver and bismuth^25,26^. To confirm that AMG in the adrenal medulla detected mercury, and to look for the presence of other toxic metals, seven-micron paraffin sections of 12 adrenal glands (8 AMG-positive, 4 AMG-negative) were subjected to LA-ICP-MS for mercury, silver, bismuth, phosphorus, aluminium, cadmium, gold, lead, chromium, nickel and iron. Analyses were carried out on an NWR-193 excimer laser hyphenated to an Agilent Technologies 7700 ICP-MS, with argon used as the carrier gas. LA-ICP-MS conditions were optimised on NIST 612 Trace Element in Glass CRM and the sample was ablated with a 50 µm spot size and a scan speed of 100 µm/s at a frequency of 20 Hz. The data were collated into a single image file using in-house developed software and visualised using FIJI.

### Ethics statement

This study (X14-029) was approved by the Human Research Committee, Sydney Local Health District (Royal Prince Alfred Hospital Zone), in accordance with the Declaration of Helsinki as revised in 2000. The institutional review board (the Human Research Committee, Sydney Local Health District) waived the need for written informed consent from relatives of individuals studied since this was a de-identified retrospective study of autopsy tissue.

## Results

### Autometallography

**Figure 2:**
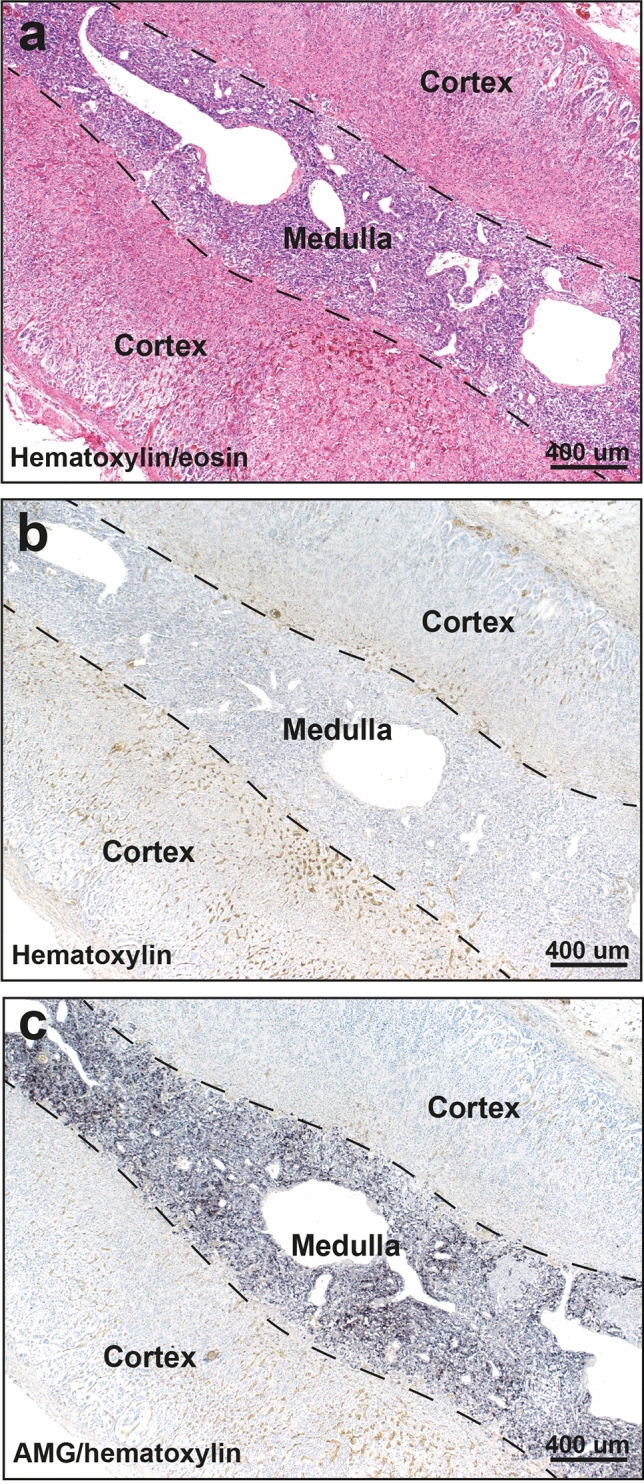
Routine and AMG staining of an AMG-positive adrenal medulla. All adjacent sections are from the same sample (from a 39 year-old male). (**a**) No histological abnormalities are seen in the adrenal cortex or medulla on hematoxylin and eosin staining. The medullary chromaffin cells have a bluish tinge. The large spaces in the medulla are empty veins. (**b**) No black grains are present in the adrenal cortex or medulla in a section stained with hematoxylin only. The yellow in the cortex shows erythrocytes. (**c**) Black AMG staining is seen in the adrenal medulla, but not in the cortex, in a section stained with AMG and hematoxylin.

**Figure 3:**
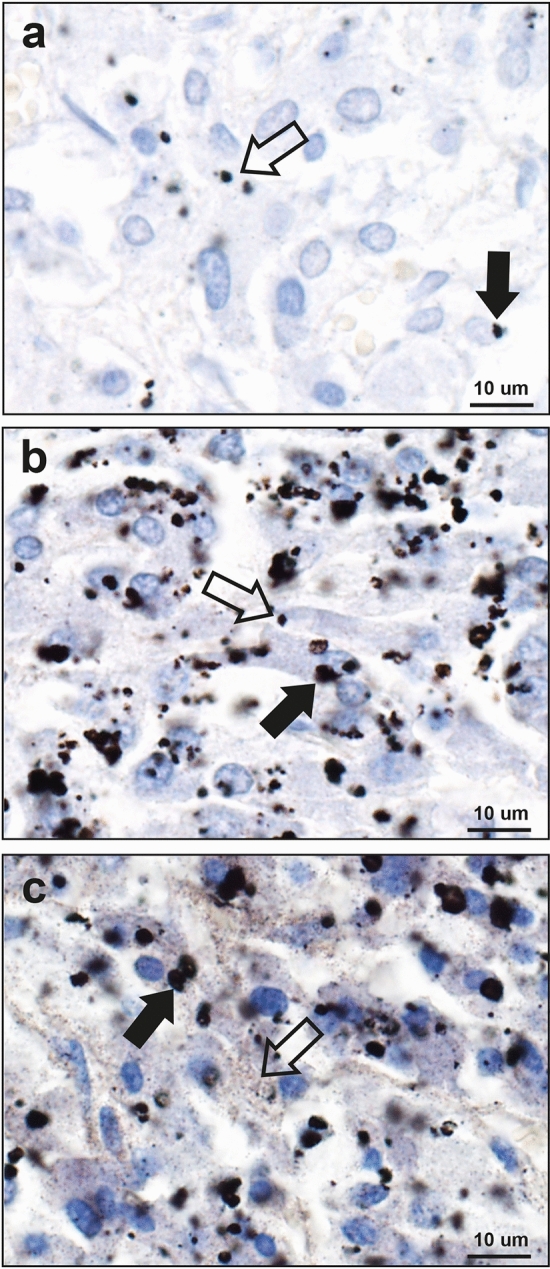
Patterns of AMG staining in the adrenal medulla. (**a**) Small dense black AMG granules, either adjacent to nuclei (filled arrow) or within the cytoplasm (open arrow), are present in fewer than 25% of these chromaffin cells (69 year-old male). (**b**) Black AMG granules, either large (filled arrow) or small (open arrow), often multiple, are present in the cytoplasm of more than 25% of chromaffin cells (32 year-old male). (**c**) Black AMG staining is present in most chromaffin cells, either as small grains dispersed in the cytoplasm (open arrow), or as these small grains accompanied by dense granules (filled arrow) (39 year-old male). AMG and hematoxylin.

Hematoxylin and eosin stained sections showed no histological abnormalities of the adrenal cortex or medulla in any samples. Hematoxylin-only stained sections showed no black grains in any adrenal cells, in contrast to the black-stained grains in the medulla of AMG-positive samples (Fig. 2). The AMG staining patterns in chromaffin cells were either: (1) single or multiple dense black granules of varying size, either adjacent to the nucleus or within the cytoplasm (the most common pattern), or (2) small black grains dispersed throughout the cytoplasm, either alone or together with dense black granules (Fig. 3). The density of AMG-containing chromaffin cells often varied across the medulla. AMG was not seen in the adrenal cortex of any samples.

**Figure 4:**
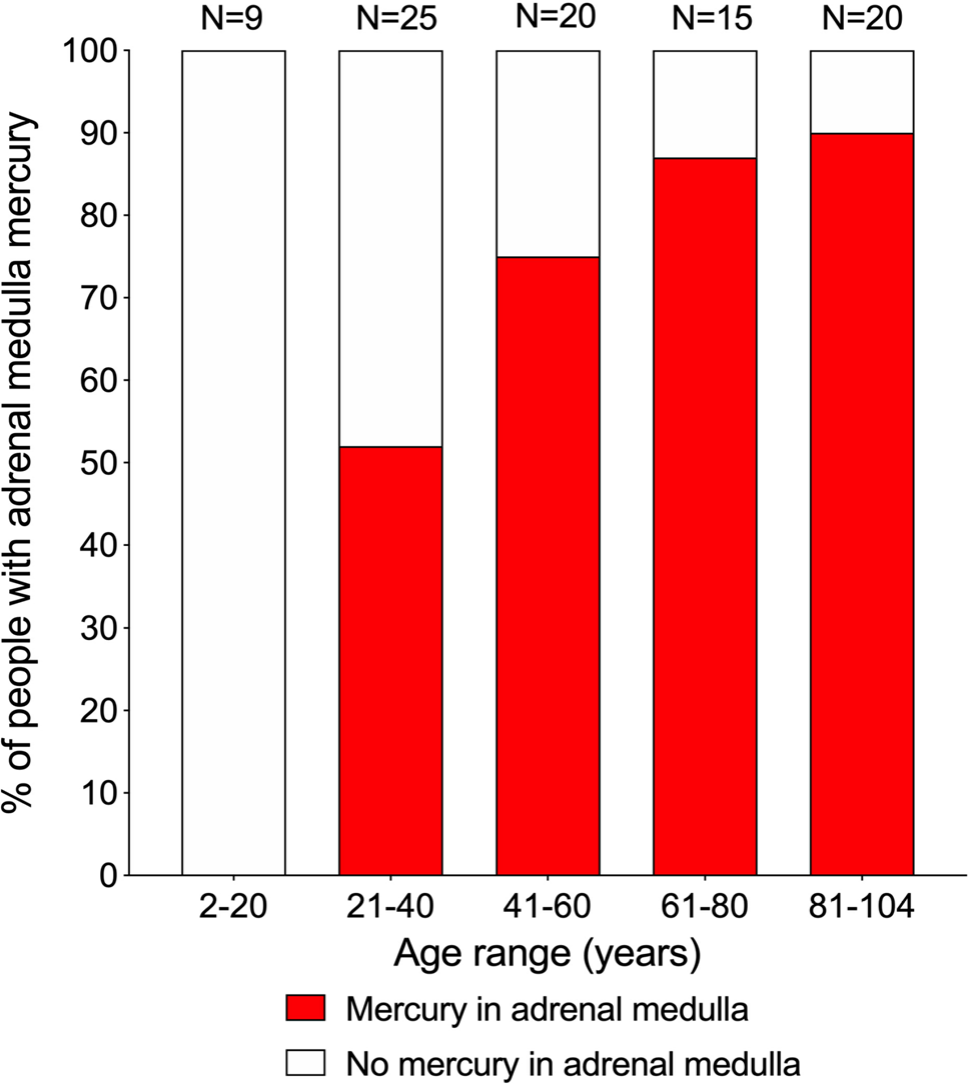
Mercury in the adrenal medulla in increasing ages. Mercury starts appearing in the adrenal medulla in the 21–40 years group, where it is present in 52% of people. The proportion of people with mercury in the medulla increases to 80% in the 41–60 years group, and reaches a maximum of 90% in the final 81–104 years group. Numbers of people in groups are above the bars.

AMG-detected mercury deposits in the adrenal medulla were present in none of the 2–20 years group, in 52% of the 21–40 years group (12% high-AMG), in 80% of the 41–60 years group (40% high-AMG), in 87% of the 61–80 years group (40% high-AMG), and in 90% of the 81–104 years group (40% high-AMG) (chi-square trend for aging p < 0.0001) (Fig. 4, Supplementary Table S1).

The 33 females in the study had a slightly higher proportion (73%) of mercury in the adrenal medulla than the 56 males (65%), probably because females were older on average than males. The proportion of people with high-AMG adrenal medulla mercury varied between the major categories of clinical diagnoses (14% for psychosis, 28% for no major clinical condition, 42% for neurodegeneration), most likely due to the different average ages in these groups (30, 52, and 74 years respectively).

### LA-ICP-MS

**Figure 5:**
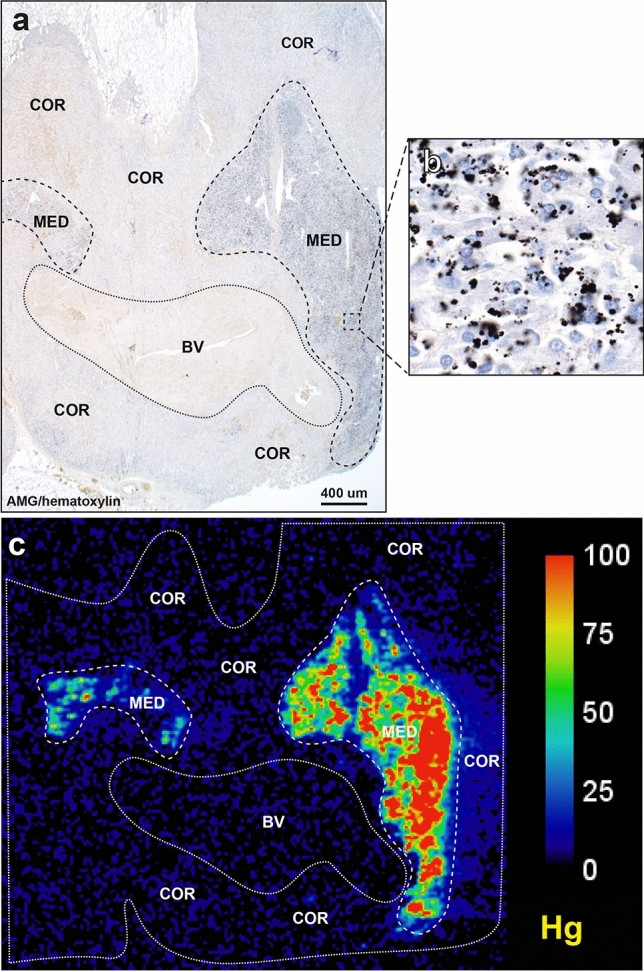
Co-localisation of mercury by AMG and LA-ICP-MS. (**a**) Black AMG staining is present in two regions of the adrenal medulla (MED), but not in the adjacent adrenal cortex (COR), or in a large blood vessel (BV). AMG and hematoxylin. (**b**) An enlargement of the area within the box in (**a**) shows numerous chromaffin cells containing black AMG grains. (**c**) LA-ICP-MS of an adjacent section shows mercury in the two adrenal medulla regions, but not in the cortex or blood vessel. All images from a 98 year-old male. Scale = counts per second (proportional to abundance).

**Figure 6:**
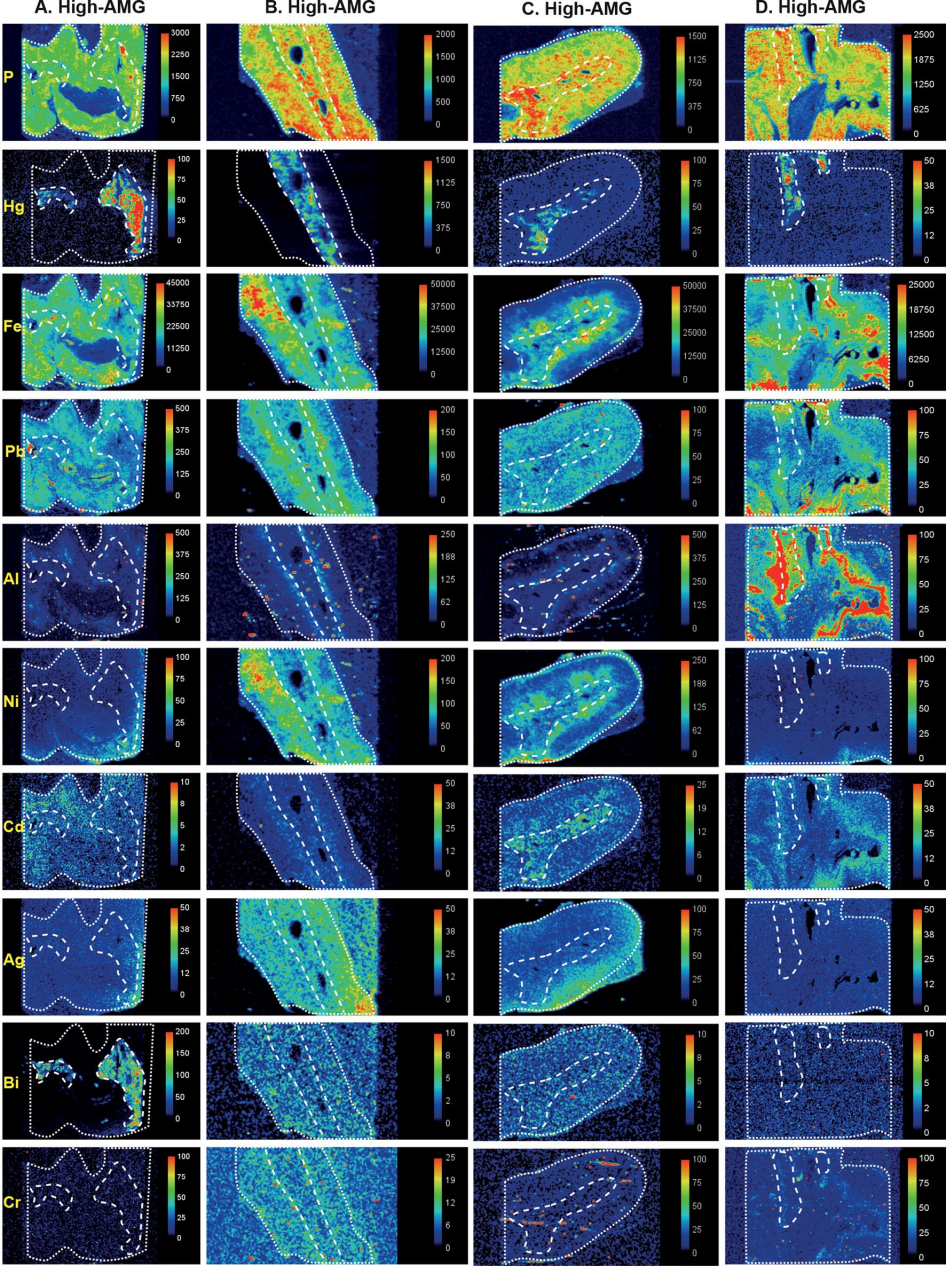
LA-ICP-MS I. The dotted lines outline the adrenal gland in the section, the medulla is outlined by the internal dashed lines, and the cortex occupies the space between the dashed and dotted lines. Phosphorus LA-ICP-MS indicates the degree of cellularity. Mercury is seen selectively in the medulla of all four samples (**A**–**D**) that had high-AMG. Metals present in both cortex and medulla are iron (**A**–**D**), lead (**A**–**D**), cadmium (**A**,**C**,**D**), bismuth (**B**,**C**), nickel (**B**,**C**), silver (**B**) and aluminium (in the cortical zona reticularis) in (**D**). Bismuth is present in the medulla in (**A**) and silver in the cortex in (**C**). Scale = counts per second (proportional to abundance).

**Figure 7:**
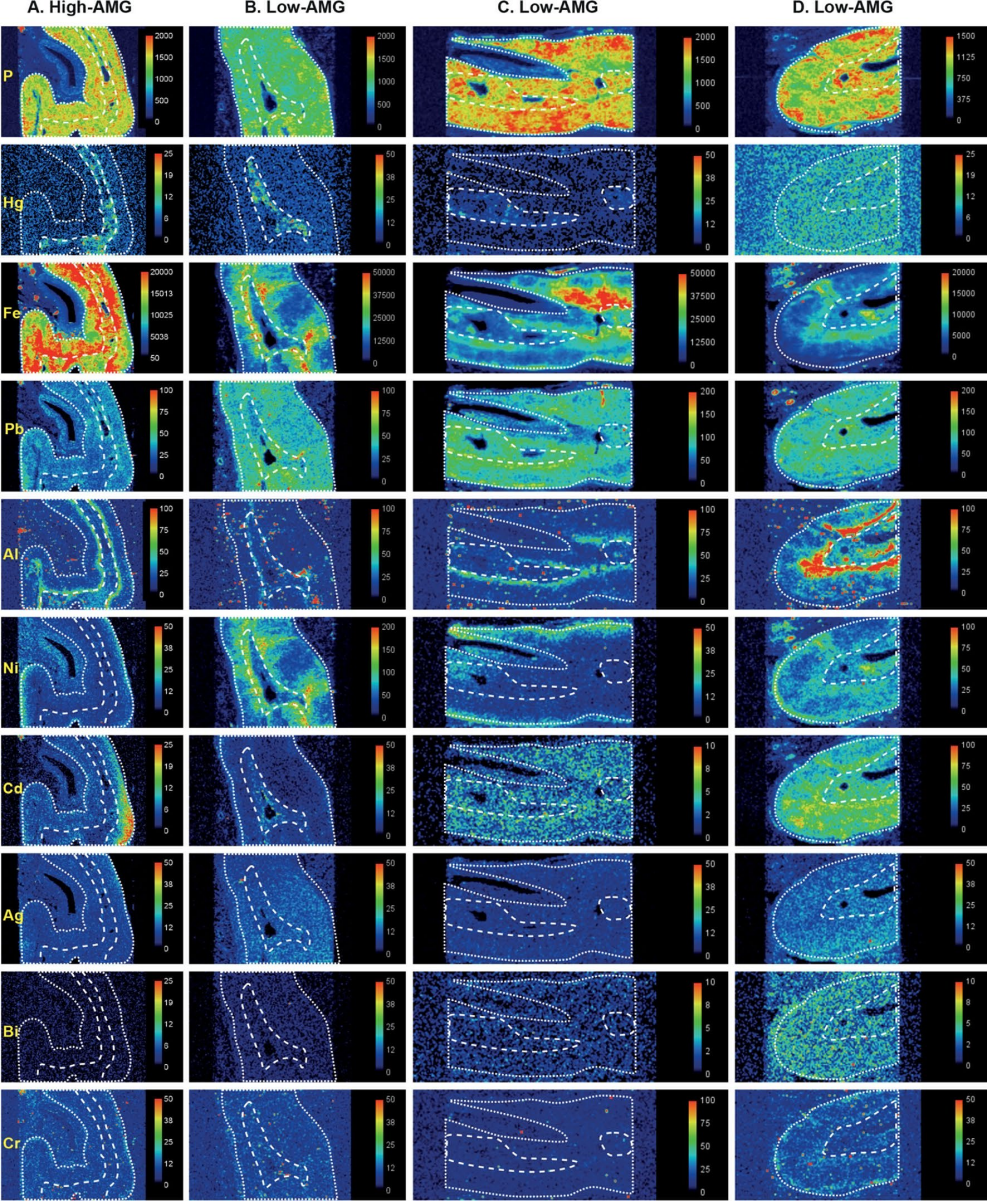
LA-ICP-MS II. The dotted lines outline the adrenal gland in the section, the medulla is outlined by the internal dashed lines, and the cortex occupies the space between the dashed and dotted lines. Phosphorus LA-ICP-MS indicates the degree of cellularity. Mercury is present selectively in the medulla of one high-AMG sample (**A**). In the three low-AMG samples, mercury is present in the medulla in two (**B**,**C**), but not detected in one (**D**). Metals present in both cortex and medulla are lead (**B**–**D**), nickel (**B**,**D**), cadmium (**C**,**D**), mercury (**D**), iron (**A**), and bismuth (**D**). Cadmium is present selectively in one medulla (**B**). Metals mostly in the cortex are iron (**B**–**D**), aluminium (in the zona reticularis, **A**,**D**), cadmium (**A**) and silver (**D**). Scale = counts per second (proportional to abundance).

**Figure 8:**
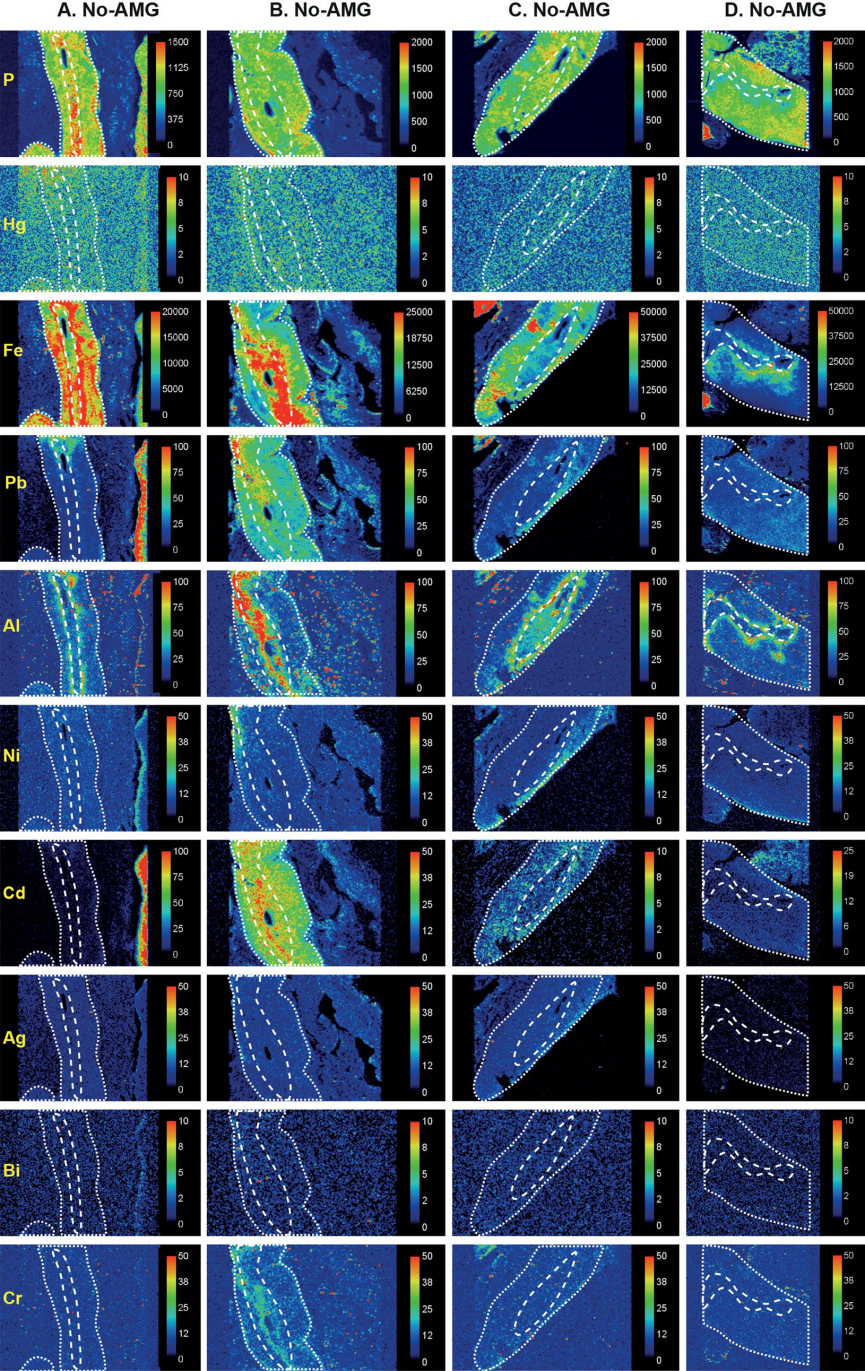
LA-ICP-MS III. The dotted lines outline the adrenal gland in the section, the medulla is outlined by the internal dashed lines, and the cortex occupies the space between the dashed and dotted lines. Phosphorus LA-ICP-MS indicates the degree of cellularity. No mercury is seen in adrenal glands that did not stain with AMG (**A**–**D**). Metals in both cortex and medulla are iron (**A**–**C**), cadmium (**B**), lead (**B**), and aluminium (in the zona reticularis, **C**). Metals present selectively in the medulla are aluminium (**B**) and chromium (**B**). Aluminium is seen mostly in the zona reticularis of the cortex in (**A**,**D**). Cadmium is present mostly in the cortex in (**C**), and iron in the inner cortex in (**D**). In (**D**), thyroid tissue (containing cadmium) is present at the upper right and lymph node tissue (containing iron) at the lower left. Scale = counts per second (proportional to abundance).

An example of the co-localisation of mercury in the adrenal medulla in both AMG and LA-ICP-MS samples is shown in Fig. 5 (from sample A in Fig. 6). The results of the LA-ICP-MS multi-elemental analyses of the adrenal glands, together with AMG staining and ages of the individuals sampled, are summarised in Table 1, and can be cross-matched with the LA-ICP-MS images in Figs. 6, 7 and 8.

**Table 1:** Potentially toxic metals found by LA-ICP-MS and AMG in 12 human adrenal glands.

Age y	Site	AMG	Hg	Fe	Pb	Al	Ni	Cd	Ag	Bi	Cr	Au	Fig
98	Medulla	++	+	+	+	−	−	+	−	+	−	−	6A
	Cortex	0	−	+	+	−	−	+	−	−	−	−	
39	Medulla	++	+	+	+	−	+	−	+	+	−	−	6B
	Cortex	0	−	+	+	−	+	−	+	+	−	−	
59	Medulla	++	+	+	+	−	−	+	−	+	−	−	6C
	Cortex	0	−	+	+	−	−	+	+	+	−	−	
46	Medulla	++	+	+	+	+	−	+	−	−	−	−	6D
	Cortex	0	−	+	+	+	−	+	−	−	−	−	
42	Medulla	++	+	+	−	−	−	+	−	−	−	−	7A
	Cortex	0	−	+	−	+	−	−	−	−	−	−	
49	Medulla	+	+	−	+	−	+	+	−	−	−	−	7B
	Cortex	0	−	+	+	−	+	−	−	−	−	−	
32	Medulla	+	+	−	+	−	+	−	−	−	−	−	7C
	Cortex	0	−	+	+	+	+	−	−	−	−	−	
41	Medulla	+	−	−	+	−	+	+	−	−	−	−	7D
	Cortex	0	−	+	+	+	+	+	+	+	−	−	
44	Medulla	0	−	+	−	−	−	−	−	−	−	−	8A
	Cortex	0	−	+	-	+	−	−	−	−	−	−	
40	Medulla	0	−	+	+	+	−	+	−	−	+	−	8B
	Cortex	0	−	+	+	−	−	+	−	−	−	−	
69	Medulla	0	−	+	−	+	−	−	−	−	−	−	8C
	Cortex	0	−	+	−	−	−	+	−	−	−	−	
32	Medulla	0	−	−	−	−	−	−	−	−	−	−	8D
	Cortex	0	−	+	−	+	−	−	−	−	−	−	

AMG: 0 none,+low, ++ high. LA-ICP-MS metals: − not detected,+detected. Fig: figure. Gold (Au) LA-ICP-MS results (all negative) are not shown in Figs. 6, 7 and 8.

In high-AMG adrenal samples, the anatomical regions where mercury was detected by AMG (corresponding to the medulla) matched those of mercury detected by LA-ICP-MS (Figs. 6, 7). In low-AMG samples, LA-ICP-MS could not detect mercury when only a few percent of chromaffin cells stained with AMG. In samples with no AMG staining, no mercury was detected on LA-ICP-MS (Fig. 8). Other toxic metals present in adrenal samples were located either in both the medulla and cortex, or in the medulla or cortex alone (Figs. 6, 7, 8). Metals detected in both the medulla and cortex were iron (N = 8), lead (N = 8), nickel (N = 4), aluminium (N = 2), and silver (N = 1). In the medulla alone, metals detected were cadmium (N = 2), aluminium (N = 2), bismuth (N = 1) and chromium (N = 1). Metals present in the cortex alone were aluminium (N = 5), iron (N = 3), and silver (N = 2). Gold was not seen in any samples.

## Discussion

The key finding of this study is that the proportion of people with mercury in their adrenal medulla increases steadily after the age of 20 years, until mercury is present in the medulla of 90% or more of people over the age of 80 years. This raises the possibility that mercury induces changes to catecholamine metabolism within the adrenal gland that contribute to the raised plasma noradrenaline and decreased adrenaline secretion found in aging.

Humans are exposed to mercury mostly from consuming mercury-containing larger fish^27^. The amount of mercury in the atmosphere is rising, due mostly to the burning of coal^28^. Atmospheric mercury enters the global atmosphere-water-soil cycle and has been found in increasing amounts in predatory fish^29^. Others sources of mercury exposure include those from occupations, mercury-containing dental amalgam restorations^27^ and from food such as rice in some countries^30^. Mercury can be detected in the chromaffin cells of primates after they have been fitted with as few as four mercury amalgam dental fillings^22^.

Our findings are paralleled by studies of the effect methylmercury has on hypertension in local communities living around Minamata Bay in Japan. Hypertension was found to be more common in the Minamata area (high methylmercury exposure) than in an area of low methylmercury exposure, and dose response trends with hair mercury levels were observed for hypertension, supporting a causal relationship between methylmercury exposure and hypertension^31^. Later work from the same group showed that mortality from hypertension was greater in Minamata city than in the whole prefecture in which Minamata is sited^32^, with further analyses supporting a link between mercury exposure and hypertension^33^.

The reason mercury preferentially localises to adrenal medulla cells is not known, but one possibility is that noradrenaline-containing cells are predisposed to take up circulating toxic metals, since noradrenergic locus ceruleus neurons in the brain stem also accumulate mercury on aging^34^. Another possibility is that chromaffin cells contain a high level of one or more of a range of metal transporters, for example, human organic anion transporter 1 or breast cancer resistance protein, that are found in other cells in the body that take up mercury, such as those of renal proximal tubules^35,36^.

Our samples were not suitable for ultrastructural studies, so we were not able to establish the subcellular location of the mercury. However, animal studies indicate that mercury in chromaffin cells is situated mostly within lysosomes, and to a lesser extent in secretory granules^22,37^. In most of our samples, AMG-stained mercury was present within compact granules in the cells, suggesting past mercury exposure with lysosomal accumulation. In a few samples, fine AMG grains were present throughout the cytoplasm, implying widespread cytoplasmic mercury from recent or ongoing exposure. This implies catecholamine metabolism could be disturbed either early after mercury exposure when cytoplasmic mercury can bind to sulfhydryl-containing proteins, or later due to disruption of lysosomal metabolic processes^38^.

Our findings, suggesting links between mercury uptake in the adrenal medulla, age-related increases in circulating noradrenaline, and hypertension, give a potential pathophysiological explanation for a number of clinical and epidemiological findings: (1) the prevalence of hypertension rises with aging^39,40^, at about the same age-related increase in the proportion of individuals in our study having mercury in their adrenal medulla. (2) Indirect evidence of sympathetic overactivity in hypertension comes from the frequent finding of tachycardia^41^ and tremor^42^ in people with raised blood pressure. (3) Hypertension has been associated with mercury exposure in people living in the vicinity of volcanoes^43,44^ (with eruptions being sources of mercury^45^), as well as in people exposed to atmospheric pollution^46,47^ that often has a mercury component^28^. (4) Hypertension and abdominal obesity are features of the metabolic syndrome, where sympathetic overdrive is implicated^5^, but why these two conditions are linked is unclear^48–50^. Several epidemiological studies report associations of circulating levels of mercury with hypertension^17,51^, obesity^52,53^, and with the metabolic syndrome^18,53,54^. The reason for this link could be accumulation of mercury in both the adrenal medulla (with increased noradrenaline output causing the hypertension) together with mercury that accumulates on aging in pituitary somatotrophs^55^, since a decrease in growth hormone levels promotes abdominal obesity^56^. (5) The onset of hypertension has been related to psychological stress^57^. Raised noradrenaline levels from mercury in chromaffin cells could cause symptoms associated with stress^58^, rather than stress being a trigger for hypertension. (6) Mercury has been suggested to underlie the hypertension seen in both mercury poisoning and in Kawasaki disease^59^.

Noradrenaline-containing cells, such as adrenal chromaffin cells and locus ceruleus neurons, take up mercury selectively, so it is possible that noradrenaline-containing sympathetic ganglion neurons also have a predilection to take up mercury, which could be a factor in the increased sympathetic nerve activity in hypertension described in humans^4^. Finding toxic metals in sympathetic ganglion neurons would imply that both nervous system and adrenal medulla-derived noradrenaline play roles in essential hypertension (Fig. 1). The paravertebral sympathetic chain is seldom removed routinely at autopsy, so a prospective autopsy study of people with and without hypertension would be needed to undertake an elemental analysis study of this type.

Metals other than mercury detected by LA-ICP-MS in the adrenal medulla included cadmium and chromium, both of which can release catecholamines from the adrenal medulla^8,60^. Lead was found in most adrenal glands, probably because of exposure to lead in petrol and domestic paints before these sources of lead were banned in Australia^61^. Iron and nickel were found mostly in the adrenal cortex, possibly because erythrocytes were more numerous in the cortex, suggesting that these were circulating metals. Silver has been found to enter the chromaffin cells of rats, where it is localised within lysosomes^62^, and silver is commonly present in noradrenergic cells of the human locus ceruleus^34^. Bismuth uptake into cells closely mirrors that of mercury^63^, but bismuth is thought to be non-toxic for humans in low doses. Bismuth use is uncommon in Australia, unlike countries such as the USA where it is often taken for gastrointestinal symptoms. The finding of multiple metals within individual adrenal medulla samples may have implications for catecholamine metabolism since harmful synergistic interactions between toxic metals, in particular mercury and cadmium, have been described^64^.

An unexpected finding was the frequent detection of aluminium in the zona reticularis of the cortex, adjacent to the medulla. Apart from its known function as a source of adrenal androgens such as dehydroepiandrosterone, important for the adrenarche, other functions of the human reticularis remain unclear, though it may be involved in xenobiotic metabolism and maintaining circadian rhythms^65^. High levels of aluminium in the reticularis could compromise these important functions. Zona reticularis cells often contain lipofuscin^66^, a wear-and-tear pigment, which is consistent with these cells having to deal with damaging xenobiotics. Exposure to aluminium phosphide, a commonly used fumigant for stored grains, can cause adrenocortical necrosis^67^, which may be because the aluminium moiety targets reticularis cells before toxic phosphine is released.

This study has several limitations. (1) This was a retrospective autopsy study, so we do not know the noradrenaline and adrenaline status of the people whose adrenal tissue we studied. We did not have access to fresh blood samples to measure catecholamine levels, or unfixed adrenal tissue to measure intra-adrenal catecholamine levels. At present, there is no imaging method of detecting toxic metals in the adrenal gland in vivo, so undertaking clinical catecholamine-adrenal mercury studies on humans is currently not possible. Substantiating links between adrenal toxic metals, catecholamine levels and hypertension would require future laboratory investigations. (2) Autometallography demonstrates inorganic mercury, but not organic mercury. However, inorganic mercury is considered to be the proximate toxic form of the metal in tissues^27^ and is therefore the most important type to measure. (3) Coronial autopsy populations, aimed largely at investigating unnatural deaths, cannot precisely replicate conditions in general populations. We tried to minimise these differences by studying people with a range of disorders, as well as those without known medical conditions who had died suddenly and unexpectedly. (4) Assessing the proportion of AMG-stained chromaffin cells was difficult, since the distribution of mercury within the medulla was often variable, chromaffin cell borders are not clearly delineated^66^, and the adrenal glands had not been dissected in a uniform way. The method used to categorise the proportion of cells containing mercury into low and high is therefore qualitative in nature. (5) We did not confirm the presence of chromaffin using histochemistry (which would have obscured AMG staining), but virtually all the cells in the human adrenal medulla are chromaffin cells, with only a few interspersed ganglion cells that are readily identified microscopically^66^. (6) In one sample, LA-ICP-MS did not detect mercury when AMG was seen in only a few chromaffin cells. This is to be expected, since autometallography, being an amplification technique, can detect nanogram amounts of inorganic mercury bound to selenide or sulphide in single cells^23^, whereas the detection limit for tissue mercury by LA-ICP-MS is 0.08 μg/g^68^.

In conclusion, mercury is found often within the adult adrenal medulla, and the proportion of people having mercury in their adrenal medulla increases on aging. The finding of mercury in the adrenal medulla provides a potential link between mercury exposure, increased sympathetic activity, and the pathogenesis of essential hypertension and the metabolic syndrome. Because of the severe health consequences of chronically raised sympathetic tone, precautionary approaches would be to limit consumption of seafood containing high mercury levels, to consider alternatives to mercury-containing amalgam fillings, to ensure that the diet contains enough selenium to help counteract the toxic effects of mercury^69^, and to take continued steps to reduce workplace exposure to mercury.

## Supplementary Information

Supplementary Information

## Data Availability

All data generated or analysed during this study are included in this published article and its Supplementary Information files.

## References

[1] Ziegler MG, Lake CR, Kopin IJ (1976). Plasma noradrenaline increases with age. Nature.

[2] Louis WJ, Doyle AE, Anavekar S (1973). Plasma norepinephrine levels in essential hypertension. N. Engl. J. Med..

[3] Goldstein DS (1983). Plasma catecholamines and essential hypertension. An analytical review. Hypertension.

[4] Guyenet PG (2006). The sympathetic control of blood pressure. Nat. Rev. Neurosci..

[5] Carnagarin R (2019). Effects of sympathetic modulation in metabolic disease. Ann. N. Y. Acad Sci.

[6] Seals DR, Esler MD (2000). Human ageing and the sympathoadrenal system. J. Physiol..

[7] Hart DT, Borowitz JL (1974). Adrenal catecholamine release by divalent mercury and cadmium. Arch. Int. Pharmacodyn. Ther..

[8] Shanbaky IO, Borowitz JL, Kessler WV (1978). Mechanisms of cadmium- and barium-induced adrenal catecholamine release. Toxicol. Appl. Pharmacol..

[9] Henningsson C, Hoffmann S, McGonigle L, Winter JS (1993). Acute mercury poisoning (acrodynia) mimicking pheochromocytoma in an adolescent. J. Pediatr..

[10] Velzeboer SC, Frenkel J, de Wolff FA (1997). A hypertensive toddler. Lancet.

[11] Wossmann W, Kohl M, Gruning G, Bucsky P (1999). Mercury intoxication presenting with hypertension and tachycardia. Arch. Dis. Child..

[12] Torres AD, Rai AN, Hardiek ML (2000). Mercury intoxication and arterial hypertension: Report of two patients and review of the literature. Pediatrics.

[13] Beck C, Krafchik B, Traubici J, Jacobson S (2004). Mercury intoxication: It still exists. Pediatr. Dermatol..

[14] Brannan EH, Su S, Alverson BK (2012). Elemental mercury poisoning presenting as hypertension in a young child. Pediatr. Emerg. Care.

[15] Evans S, Smith J, Caron E (2018). A case of mercury toxicity complicated by acute inflammatory demyelinating polyneuropathy. J. Child. Neurol..

[16] Yan J, Pan Y, Tang Z, Song Y (2019). Mercury poisoning presenting with hypertension: Report of 2 cases. Am. J. Med. Genet. B Neuropsychiatr. Genet..

[17] Hu XF, Singh K, Chan HM (2018). Mercury exposure, blood pressure, and hypertension: A systematic review and dose-response meta-analysis. Environ. Health Perspect..

[18] Roy C, Tremblay PY, Ayotte P (2017). Is mercury exposure causing diabetes, metabolic syndrome and insulin resistance? A systematic review of the literature. Environ. Res..

[19] Berlin M, Ullberg S (1963). Accumulation and retention of mercury in the mouse. II. An autoradiographic comparison of phenylmercuric acetate with inorganic mercury. Arch. Environ. Health.

[20] Kozma L, Papp L, Varga EE, Gomba S (1996). Accumulation of Hg(II) Ions in Mouse Adrenal Gland. Pathol. Oncol. Res..

[21] Khayat A, Dencker L (1984). Organ and cellular distribution of inhaled metallic mercury in the rat and Marmoset monkey (*Callithrix jacchus*): Influence of ethyl alcohol pretreatment. Acta. Pharmacol. Toxicol. (Copenh.).

[22] Danscher G, Horsted-Bindslev P, Rungby J (1990). Traces of mercury in organs from primates with amalgam fillings. Exp. Mol. Pathol..

[23] Danscher G, Moller-Madsen B (1985). Silver amplification of mercury sulfide and selenide: A histochemical method for light and electron microscopic localization of mercury in tissue. J. Histochem. Cytochem..

[24] Pamphlett R, Png FY (1998). Shrinkage of motor axons following systemic exposure to inorganic mercury. J. Neuropathol. Exp. Neurol..

[25] Danscher G, Stoltenberg M, Juhl S (1994). How to detect gold, silver and mercury in human brain and other tissues by autometallographic silver amplification. Neuropathol. Appl. Neurobiol..

[26] Danscher G, Stoltenberg M, Kemp K, Pamphlett R (2000). Bismuth autometallography: Protocol, specificity, and differentiation. J. Histochem. Cytochem..

[27] Clarkson TW (1997). The toxicology of mercury. Crit. Rev. Clin. Lab. Sci..

[28] Streets DG (2011). All-time releases of mercury to the atmosphere from human activities. Environ. Sci. Technol..

[29] Schartup AT (2019). Climate change and overfishing increase neurotoxicant in marine predators. Nature.

[30] Zhao H (2019). Mercury contents in rice and potential health risks across China. Environ. Int..

[31] Yorifuji T, Tsuda T, Kashima S, Takao S, Harada M (2010). Long-term exposure to methylmercury and its effects on hypertension in Minamata. Environ. Res..

[32] Inoue S, Yorifuji T, Tsuda T, Doi H (2012). Short-term effect of severe exposure to methylmercury on atherosclerotic heart disease and hypertension mortality in Minamata. Sci. Total Environ..

[33] Yorifuji T, Tsuda T (2016). Epidemiological studies of neurological signs and symptoms and blood pressure in populations near the industrial methylmercury contamination at Minamata, Japan. Arch. Environ. Occup. Health.

[34] Pamphlett R, Bishop DP, Kum Jew S, Doble PA (2018). Age-related accumulation of toxic metals in the human locus ceruleus. PLoS One.

[35] Zalups RK, Aslamkhan AG, Ahmad S (2004). Human organic anion transporter 1 mediates cellular uptake of cysteine-S conjugates of inorganic mercury. Kidney Int..

[36] Bridges CC, Zalups RK, Joshee L (2015). Toxicological significance of renal Bcrp: Another potential transporter in the elimination of mercuric ions from proximal tubular cells. Toxicol. Appl. Pharmacol..

[37] Rasmussen BL, Thorlacius-Ussing O (1987). Ultrastructural localization of mercury in adrenals from rats exposed to methyl mercury. Virchows Arch. B Cell. Pathol. Incl. Mol. Pathol..

[38] Lawrence RE, Zoncu R (2019). The lysosome as a cellular centre for signalling, metabolism and quality control. Nat. Cell Biol..

[39] Benjamin EJ (2019). Heart disease and stroke statistics-2019 update: A report from the American Heart Association. Circulation.

[40] Kit BK (2015). Prevalence of and trends in dyslipidemia and blood pressure among US children and adolescents, 1999–2012. JAMA Pediatr..

[41] Palatini P (1997). Relationship of tachycardia with high blood pressure and metabolic abnormalities: A study with mixture analysis in three populations. Hypertension.

[42] Hu T (2016). Postural hand tremor and incident hypertension in young to middle-aged adults: The Bogalusa heart study. J. Hypertens..

[43] Longo BM (2013). Adverse health effects associated with increased activity at Kilauea Volcano: A repeated population-based survey. ISNR Public Health.

[44] Brook RD, Brook JR, Tam EK (2019). Volcanic smog and cardiometabolic health: Hawaiian hypertension?. J. Clin. Hypertens. (Greenwich).

[45] Varekamp JC, Buseck PR (1986). Global mercury flux from volcanic and geothermal sources. J. Appl. Geochem..

[46] Brook RD (2016). Extreme air pollution conditions adversely affect blood pressure and insulin resistance: The air pollution and cardiometabolic disease study. Hypertension.

[47] Dong GH (2013). Association between long-term air pollution and increased blood pressure and hypertension in China. Hypertension.

[48] Yanai H (2008). The underlying mechanisms for development of hypertension in the metabolic syndrome. Nutr. J..

[49] Mendizabal Y, Llorens S, Nava E (2013). Hypertension in metabolic syndrome: Vascular pathophysiology. Int. J. Hypertens..

[50] Mule G, Calcaterra I, Nardi E, Cerasola G, Cottone S (2014). Metabolic syndrome in hypertensive patients: An unholy alliance. World J. Cardiol..

[51] da CunhaMartins Jr A, Carneiro MFH, Grotto D, Adeyemi JA, Barbosa F (2018). Arsenic, cadmium, and mercury-induced hypertension: Mechanisms and epidemiological findings. J. Toxicol. Environ. Health B Crit. Rev..

[52] Park JS, Ha KH, He K, Kim DJ (2017). Association between blood mercury level and visceral adiposity in adults. Diabetes Metab. J..

[53] Lee K (2018). Blood mercury concentration in relation to metabolic and weight phenotypes using the KNHANES 2011–2013 data. Int. Arch. Occup. Environ. Health.

[54] Planchart A, Green A, Hoyo C, Mattingly CJ (2018). Heavy metal exposure and metabolic syndrome: Evidence from human and model system studies. Curr. Environ. Health Rep..

[55] Pamphlett R, Kum Jew S, Doble PA, Bishop DP (2019). Elemental analysis of aging human pituitary glands implicates mercury as a contributor to the somatopause. Front. Endocrinol. (Lausanne).

[56] Rudman D (1991). Effects of human growth hormone on body composition in elderly men. Horm. Res..

[57] Liu MY, Li N, Li WA, Khan H (2017). Association between psychosocial stress and hypertension: A systematic review and meta-analysis. Neurol. Res..

[58] Kantorovich V, Eisenhofer G, Pacak K (2008). Pheochromocytoma: An endocrine stress mimicking disorder. Ann. N. Y. Acad. Sci..

[59] Mutter J, Yeter D (2008). Kawasaki's disease, acrodynia, and mercury. Curr. Med. Chem..

[60] Liu PS, Lin MK (1997). Biphasic effects of chromium compounds on catecholamine secretion from bovine adrenal medullary cells. Toxicology.

[61] Daley GM, Pretorius CJ, Ungerer JP (2018). Lead toxicity: An Australian PERSPECTIVE. Clin. Biochem. Rev..

[62] Rungby J (1986). Exogenous silver in dorsal root ganglia, peripheral nerve, enteric ganglia, and adrenal medulla. Acta Neuropathol..

[63] Ross JF, Switzer RC, Poston MR, Lawhorn GT (1996). Distribution of bismuth in the brain after intraperitoneal dosing of bismuth subnitrate in mice: Implications for routes of entry of xenobiotic metals into the brain. Brain Res..

[64] Cobbina SJ (2015). Interaction of four low dose toxic metals with essential metals in brain, liver and kidneys of mice on sub-chronic exposure. Environ. Toxicol. Pharmacol..

[65] Vinson GP (2016). Functional zonation of the adult mammalian adrenal cortex. Front. Neurosci..

[66] Carney JA, Mills SE (2012). Histology for Pathologists.

[67] Chugh SN (1989). Adrenocortical involvement in aluminium phosphide poisoning. Indian J. Med. Res..

[68] Niehoff AC (2015). Quantitative bioimaging to investigate the uptake of mercury species in *Drosophila melanogaster*. Anal. Chem..

[69] Ralston NV, Raymond LJ (2010). Dietary selenium's protective effects against methylmercury toxicity. Toxicology.

