# Supplementary material for: Mercury in the human adrenal medulla could contribute to increased plasma noradrenaline in aging
Source: Sci Rep. 2021 Feb 3;11:2961. doi: 10.1038/s41598-021-82483-y (PMC7858609; doi:10.1038/s41598-021-82483-y)
Supplement: Supplementary file 1 — Supplementary Information [file 41598_2021_82483_MOESM1_ESM.pdf]

Mercury in the human adrenal medulla could contribute to increased plasma noradrenaline in aging

Roger Pamphlett[1,2], Stephen Kum Jew[1], Philip A. Doble[3], David P. Bishop[3]

[1]Discipline of Pathology, Sydney Medical School, Brain and Mind Centre, The University of Sydney, Sydney, New South Wales, Australia

[2]Department of Neuropathology, Royal Prince Alfred Hospital, Sydney, New South Wales, Australia

[3]Elemental Bio-Imaging Facility, School of Mathematical and Physical Sciences, University of Technology Sydney, Sydney, New South Wales, Australia

Supplementary Table 1. Age range, gender, clinical history, cause of death, and adrenal medulla autometallography (AMG) of individuals.

ID	Age range (years)	Gender	Clinical	Cause of death	AMG
#01	2 to 20	Male	None	Drowning	0
#02	2 to 20	Female	None	Trauma	0
#03	2 to 20	Male	None	Trauma	0
#04	2 to 20	Female	None	Respiratory	0
#05	2 to 20	Male	None	Suicide	0
#06	2 to 20	Female	None	Trauma	0
#07	2 to 20	Male	Psychosis	Suicide	0
#08	2 to 20	Male	Psychosis	Suicide	0
#09	2 to 20	Male	None	Trauma	0
#10	21 to 40	Male	None	Drowning	0
#11	21 to 40	Female	Epilepsy	Undetermined	0
#12	21 to 40	Female	None	Suicide	+
#13	21 to 40	Male	Psychosis	Suicide	0
#14	21 to 40	Male	Psychosis	Infection	0
#15	21 to 40	Male	Neurodegeneration	Suicide	++
#16	21 to 40	Female	Psychosis	Suicide	0
#17	21 to 40	Male	Psychosis	Drug overdose	0
#18	21 to 40	Male	None	Suicide	+
#19	21 to 40	Male	Psychosis	Trauma	0
#20	21 to 40	Male	PTSD	Drug overdose	0
#21	21 to 40	Male	None	Suicide	++
#22	21 to 40	Male	Psychosis	Trauma	+
#23	21 to 40	Female	Down syndrome	Drug overdose	0
#24	21 to 40	Female	None	Drowning	+
#25	21 to 40	Male	None	Drug overdose	+
#26	21 to 40	Male	Psychosis	Cardiovascular	0
#27	21 to 40	Female	Epilepsy	Drowning	+
#28	21 to 40	Male	Psychosis	Suicide	0
#29	21 to 40	Female	Anorexia nervosa	Undernutrition	++
#30	21 to 40	Male	None	Drowning	++

ID	Age range (years)	Gender	Clinical	Cause of death	AMG
#31	21 to 40	Male	None	Suicide	+
#32	21 to 40	Female	None	Suicide	0
#33	21 to 40	Female	Psychosis	Suicide	+
#34	21 to 40	Male	None	Trauma	+
#35	41 to 60	Male	Psychosis	Drug overdose	+
#36	41 to 60	Male	None	Suicide	0
#37	41 to 60	Female	Psychosis	Suicide	+
#38	41 to 60	Male	None	Suicide	++
#39	41 to 60	Male	Psychosis	Drug overdose	0
#40	41 to 60	Male	Psychosis	Drug overdose	0
#41	41 to 60	Female	None	Drowning	++
#42	41 to 60	Male	Psychosis	Respiratory	0
#43	41 to 60	Male	Psychosis	Drug overdose	+
#44	41 to 60	Male	None	Suicide	+
#45	41 to 60	Female	Psychosis	Drug overdose	++
#46	41 to 60	Male	None	Drug overdose	+
#47	41 to 60	Female	Psychosis	Suicide	++
#48	41 to 60	Male	Neurodegeneration	Undetermined	++
#49	41 to 60	Male	Psychosis	Undetermined	+
#50	41 to 60	Female	Psychosis	Cirrhosis	+
#51	41 to 60	Male	Neurodegeneration	Undetermined	0
#52	41 to 60	Female	None	Drowning	++
#53	41 to 60	Male	Neurodegeneration	Suicide	++
#54	41 to 60	Male	None	Suicide	+
#55	61 to 80	Male	Neurodegeneration	Choking	+
#56	61 to 80	Male	Neurodegeneration	Choking	+
#57	61 to 80	Male	Psychosis	Suicide	++
#58	61 to 80	Male	None	Trauma	+
#59	61 to 80	Male	Neurodegeneration	Choking	++
#60	61 to 80	Female	Neurodegeneration	Suicide	++
#61	61 to 80	Male	Neurodegeneration	Drowning	0
#62	61 to 80	Male	Neurodegeneration	Cardiovascular	+
#63	61 to 80	Male	Neurodegeneration	Cardiovascular	+
#64	61 to 80	Female	Neurodegeneration	Infection	++
#65	61 to 80	Female	Neurodegeneration	Trauma	0
#66	61 to 80	Female	Neurodegeneration	Cardiovascular	++
#67	61 to 80	Male	Neurodegeneration	Cardiovascular	++
#68	61 to 80	Female	Neurodegeneration	Trauma	+
#69	61 to 80	Female	Neurodegeneration	Infection	+
#70	81 to 104	Male	Neurodegeneration	Cerebrovascular	+
#71	81 to 104	Male	Neurodegeneration	Choking	++
#72	81 to 104	Female	Neurodegeneration	Cardiovascular	+
#73	81 to 104	Male	Neurodegeneration	Cardiovascular	++
#74	81 to 104	Female	Neurodegeneration	Cardiovascular	+
#75	81 to 104	Female	None	Cardiovascular	0
#76	81 to 104	Male	None	Cardiovascular	++
#77	81 to 104	Male	Neurodegeneration	Trauma	+
#78	81 to 104	Female	Neurodegeneration	Trauma	+
#79	81 to 104	Male	Neurodegeneration	Cardiovascular	++

ID	Age range (years)	Gender	Clinical	Cause of death	AMG
#80	81 to 104	Male	None	Infection	++
#81	81 to 104	Female	Neurodegeneration	Cardiovascular	+
#82	81 to 104	Female	None	Suicide	+
#83	81 to 104	Female	None	Trauma	+
#84	81 to 104	Male	None	Trauma	+
#85	81 to 104	Male	None	Trauma	++
#86	81 to 104	Male	None	Cancer	+
#87	81 to 104	Male	None	Cerebrovascular	0
#88	81 to 104	Female	None	Cardiovascular	++
#89	81 to 104	Female	None	Cardiovascular	++

Adrenal medulla AMG: 0 none, + low, ++ high. ID: identity number
